# Prion strain-dependent tropism is maintained between spleen and granuloma and relies on lymphofollicular structures

**DOI:** 10.1038/s41598-019-51084-1

**Published:** 2019-10-10

**Authors:** Iman Al-Dybiat, Mohammed Moudjou, Davy Martin, Fabienne Reine, Laetitia Herzog, Sandrine Truchet, Patricia Berthon, Hubert Laude, Human Rezaei, Olivier Andréoletti, Vincent Béringue, Pierre Sibille

**Affiliations:** 1 VIM, INRA, Université Paris-Saclay, 78350 Jouy-en-Josas, France; 2 UMR Infectiologie et Santé Publique, 37380 Nouzilly, France; 3 UMR INRA ENVT 1225, Interactions Hôtes Agents Pathogènes, Ecole Nationale Vétérinaire de Toulouse, 23 Chemin des Capelles, 31076 Toulouse, France

**Keywords:** Immunological models, Infection

## Abstract

In peripherally acquired prion diseases, prions move through several tissues of the infected host, notably in the lymphoid tissue, long before the occurrence of neuroinvasion. Accumulation can even be restricted to the lymphoid tissue without neuroinvasion and clinical disease. Several experimental observations indicated that the presence of differentiated follicular dendritic cells (FDCs) in the lymphoid structures and the strain type are critical determinants of prion extraneural replication. In this context, the report that granulomatous structures apparently devoid of FDCs could support prion replication raised the question of the requirements for prion lymphotropism. The report also raised the possibility that nonlymphoid tissue-tropic prions could actually target these inflammatory structures. To investigate these issues, we examined the capacity of closely related prions, albeit with opposite lymphotropism (or FDC dependency), for establishment in experimentally-induced granuloma in ovine PrP transgenic mice. We found a positive correlation between the prion capacity to accumulate in the lymphoid tissue and granuloma, regardless of the prion detection method used. Surprisingly, we also revealed that the accumulation of prions in granulomas involved lymphoid-like structures associated with the granulomas and containing cells that stain positive for PrP, Mfge-8 but not CD45 that strongly suggest FDCs. These results suggest that the FDC requirement for prion replication in lymphoid/inflammatory tissues may be strain-dependent.

## Introduction

Prions cause neurodegenerative and fatal disorders resulting from the conversion of the host-encoded cellular prion protein PrP^C^ into a pathological misfolded protein termed PrP^Sc^. Human prion diseases can be of genetic, sporadic or infectious origin^1^. In farm animals, prions have been responsible for the bovine spongiform encephalopathy (BSE) epidemic that arose mainly in Great Britain and western Europe in the 1990s and ultimately adapted to humans as the variant Creutzfeldt-Jacob disease (vCJD)^2^. Scrapie in sheep and goats and chronic wasting disease (CWD) in cervids, which has recently reached the European countries^3^, are other forms of prion diseases in animals. Multiple conformers of PrP^Sc^ or strains of prions are recognized in the same host species, exhibiting specific incubation periods, a stereotyped neuropathology, and specific tropism for the lymphoid tissue (for a review^4^). Colonization of secondary lymphoid organs such as spleen, lymph nodes, gut-associated lymphoid tissue and tonsils occurs long before neuroinvasion following peripheral infection by so-called lymphoid tissue tropic (LTT) prions^5–7^. Such extraneural prion replication has been observed in natural cases of sheep scrapie, CWD, humans infected with vCJD and in many experimental rodent models of prion disease. Lymphoid cell participation in this process has been extensively studied using mice with constitutive or induced deficits in specific cell populations^8^. A large body of experimental evidence indicates that follicular dendritic cells (FDCs) play a key role in prion replication in lymphoid tissues. FDCs have been identified as the first site of PrP^Sc^ accumulation in lymphoid tissues^9,10^, and the restriction of PrP^C^ expression to FDCs is sufficient to sustain prion replication in the spleen^11–13^. The absence or dedifferentiation of FDCs or ablation of PrP^C^ expression in FDCs is mostly associated with the absence of prion replication in the spleen^11,12,14,15^, but not in the lymph nodes^16^. These contrasted findings suggested that despite their resemblance, there were features that drive striking different behaviors between both lymphoid structures. In addition, tertiary lymphoid organs may also develop under chronic inflammation stimuli and have been described to replicate prion ectopically in structures containing FDC-positive lymphoid follicles^17,18^.

The recent finding that the prion species barrier is lower in lymphoid tissues than in the central nervous system (CNS)^19^ and that BSE prions responsible for vCJD can circulate unnoticed in the lymphoid system of exposed humans with a higher prevalence than normally expected^20^ has raised the need to identify factors that can affect prion replication in lymphoid or inflammatory organs. In this context, the observations of Heikenwalder and colleagues that granulomatous structures devoid of FDCs were able to replicate prions in experimental mouse models of prion diseases was puzzling^21^, raising the possibility that such structures may exhibit some permissiveness to LTT toward non-LTT prions. To address this issue, we studied the capacity of prions with opposite lymphotropism to accumulate in the spleen and granulomatous structures and brains of ovine PrP transgenic mice. We demonstrated that prion tropism was mostly conserved between the spleen and granuloma. We further showed that prions mainly accumulated in lymphofollicular structures and were associated with both FDCs (identified as PrP^+^, mfge-8^+^ CD45^−^ stromal cells) and CD68^+^ macrophages in the lymphofollicular structures surrounding the granuloma.

## Results

### Experimental setup and description of the prion strains used

**Figure 1:**
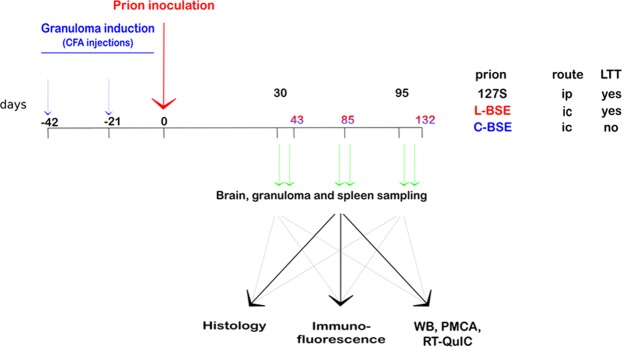
Experimental setup. At 42 and 21 days before prion inoculation, the mice were injected subcutaneously with a PBS/CFA emulsion. At day 0, the mice were inoculated with brain homogenate from terminally ill mice infected with either 127 S scrapie (intraperitoneal inoculation), L- or C-BSE (both by intracerebral inoculation, see results section). For each prion inoculum, brain, granuloma and spleen were collected at various days post-inoculation, and samples were processed for either histology, immunofluorescence, western blot or PMCA/RT-QuIC *in vitro* prion amplification.

For granuloma induction, ovine PrP transgenic mice (tg338 line^22^) were treated with two successive subcutaneous injections of Complete Freund’s Adjuvant (CFA) emulsified in phosphate-buffered saline, approximately 40 days and 20 days before prion challenge (Fig. 1)^21^. The granulomas were clearly detectable by eye or palpation on the back of the mice from the time of prion inoculation until the terminal stage of disease.

To calibrate the experiment, granulomatous tg338 mice were inoculated with 127 S scrapie prions via the intraperitoneal (ip) route. The 127 S is an LTT prion that induces short, prototypal disease in tg338 mice^23^. Half of the inoculated mice were euthanized at day 30 post-infection, at a time when PrP^res^ is easily detected in the spleen^23^. The other mice were euthanized at near the terminal disease stage, at day 95.

**Figure 2:**
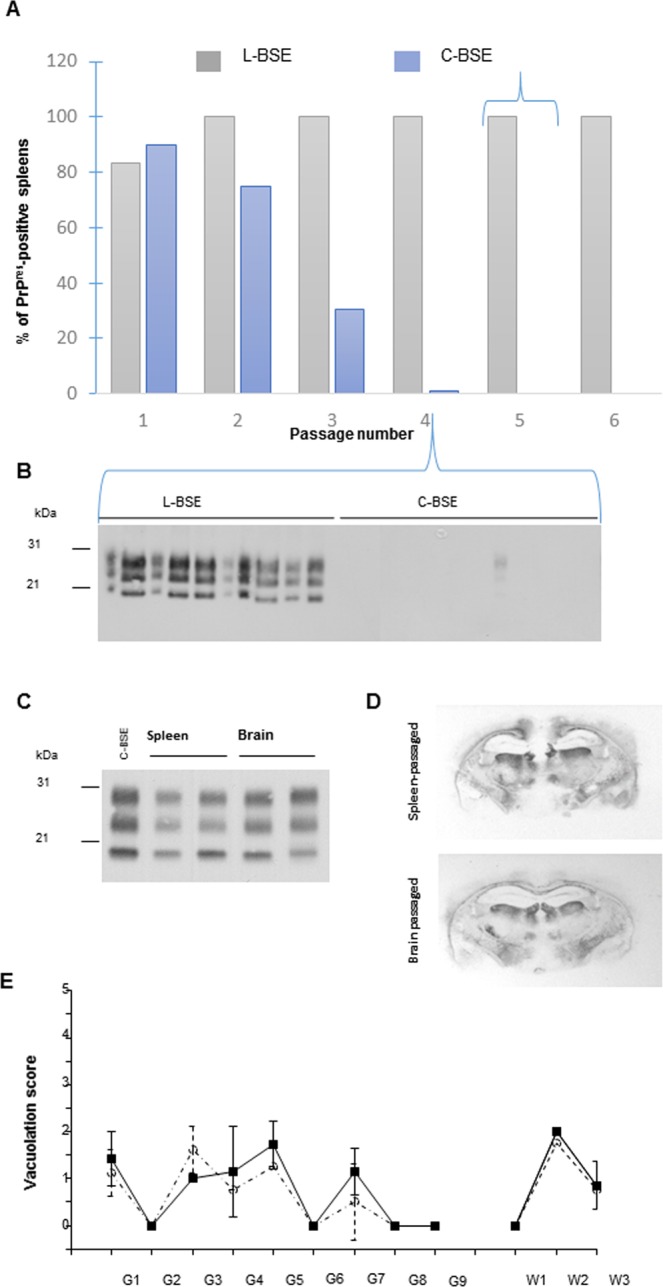
L-BSE and C-BSE prions exhibit distinct tropisms for the splenic tissue of tg338 mice. (**A**) The proportions of PrP^res^-positive spleens during iterative transmission of L-BSE (2 isolates, designated BASE and L-BSE (Fr7)) and C-BSE sources (6 isolates^24^) in tg338 mice. (**B**) Western blot analysis at the fourth passage illustrating the differences in PrP^res^ accumulation levels in the spleen. (**C**–**E**) tg338 spleen-passaged L-BSE prions share the same phenotypic characteristics as brain-passaged L-BSE prions in tg338 mice. The PrP^res^ electrophoretic pattern (**C**), PrP^res^ distribution (representative histoblot at the level of the hippocampus, (**D**) and vacuolation profile (**E**) are similar. Mean ± SEM (n = 3 mice) scores reflecting the intensity of vacuolation in tg338 mice inoculated with brain material (plain line, square symbol) or spleen material (dotted line, circle symbol). Standard brain areas in gray (G) matter and in white (W) matter areas: G1, dorsal medulla; G2, cerebellar cortex; G3, superior colliculus; G4, hypothalamus; G5, medial thalamus; G6, hippocampus; G7, septum; G8, medial cerebral cortex at the level of the thalamus; G9, medial cerebral cortex at the level of the septum; W1, cerebellar white matter; W2, white matter of the mesencephalic tegmentum; and W3, pyramidal tract.

In the second set of experiments, two closely related prions, derived from the adaptation of classical BSE (C-BSE) and atypical L-type BSE (L-BSE) to ovinized tg338 mice^24^, were inoculated to granulomatous tg338 mice. Once terminally adapted to the tg338 host, C-BSE and L-BSE prions display similar biochemical and biological features^24^, except opposite tropism for the lymphoid tissue, as shown in Fig. 2. Western blot analysis for the presence of PrP^res^ indicated that the capacity of C-BSE prions to establish in the spleen was lost on serial passage, whereas it was preserved for L-BSE prions (Fig. 2A). At the 4^th^ passage, all the spleens from L-BSE infected animals were positive for PrP^res^ (Fig. 2B). Conversely, the spleens collected from C-BSE infected animals were hardly positive. At the 4^th^ passage, quantification of infectivity by an incubation time bioassay indicated that the spleens of mice infected with C-BSE prions that were PrP^res^ negative were over 10^4^-fold less infectious than the spleens of mice infected with L-BSE prions (Table 1). Worthy of note, spleen and brain material from L-BSE infected mice induced equivalent strain phenotype in reporter tg338 mice with regard to incubation time (Table 1), PrP^res^ electrophoretic pattern (Fig. 2C), PrP^res^ distribution in the hippocampus (Fig. 2D) and vacuolation distribution in the brain (Fig. 2E). The patterns of PrP^res^ and vacuolization distribution were superimposable to that of C-BSE in these mice^24^. Therefore, there was no divergent evolution of L-BSE prions on passage through spleen compared to brain.

**Table 1:** Mean survival time of tg338 reporter mice inoculated with spleen material from tg338 mice infected with L-BSE or C-BSE.

tissue	Dilution	L-BSE		C-BSE	
		n/n0	Mean survival time* (days ± sem)	n/n0	Mean survival time* (days ± sem)
spleen	1/1	6/6	144 ± 2^‡^	4/4	229 ± 4^§^
	1/1	6/6	142 ± 2^‡^	6/6	138 ± 1
	10-4	6/6	na	6/6	176 ± 3
brain	10-5	6/6	261 ± 16	6/6	217 ± 9^§^
	10-6	6/6	281; 307	2/6	286;534
	10-7	0/6	>660	0/6	>660

^*^Mean survival time of tg338 reporter mice inoculated with spleen material from tg338 infected with L-BSE or C-BSE.

^‡,§^For pair comparison: this indicates that spleens of L-BSE infected animal are 1000 to 10000-fold more infectious than those of C-BSE infected animals. The symbols indicate inoculum dilution factors for which the incubation times (i.e. the inoculum concentration) are similar.

For the inoculation of granulomatous tg338 mice, brain extracts of both L-BSE and C-BSE prions from the 5^th^ passage in tg338 mice (i.e., stabilized L- and C-BSE strains) were used^24^. Intracerebral inoculation was performed because the time to disease after intraperitoneal inoculation would have exceeded the expected resolution time of the granuloma. In addition, at the dose used, intracerebral infection can be considered as both intracerebral and intravenous infection, due to inoculum spillover in the blood stream. Consequently, this route always targets the spleen in addition to the CNS as efficiently and within the same time window as an intraperitoneal route^23^. A proportion of mice were euthanized at 2 intermediate time points at 1/3 (day 43) and 2/3 (day 85) of the incubation time and approx. 2 weeks before the estimated terminal stage of disease, at day 132 post-inoculation. As controls for studying prion remanence in the lymphoid and granulomatous structures, PrP^0/0^ mice, in which granulomatous structures were induced, were inoculated with L-BSE and C-BSE prions and euthanized at 43 dpi.

Brains, spleens and granulomas were collected and processed for PrP^Sc^ detection using either histological or biochemical techniques (detailed in the methods section).

### Time course accumulation of 127 S scrapie prions in the granuloma and spleen

**Figure 3:**
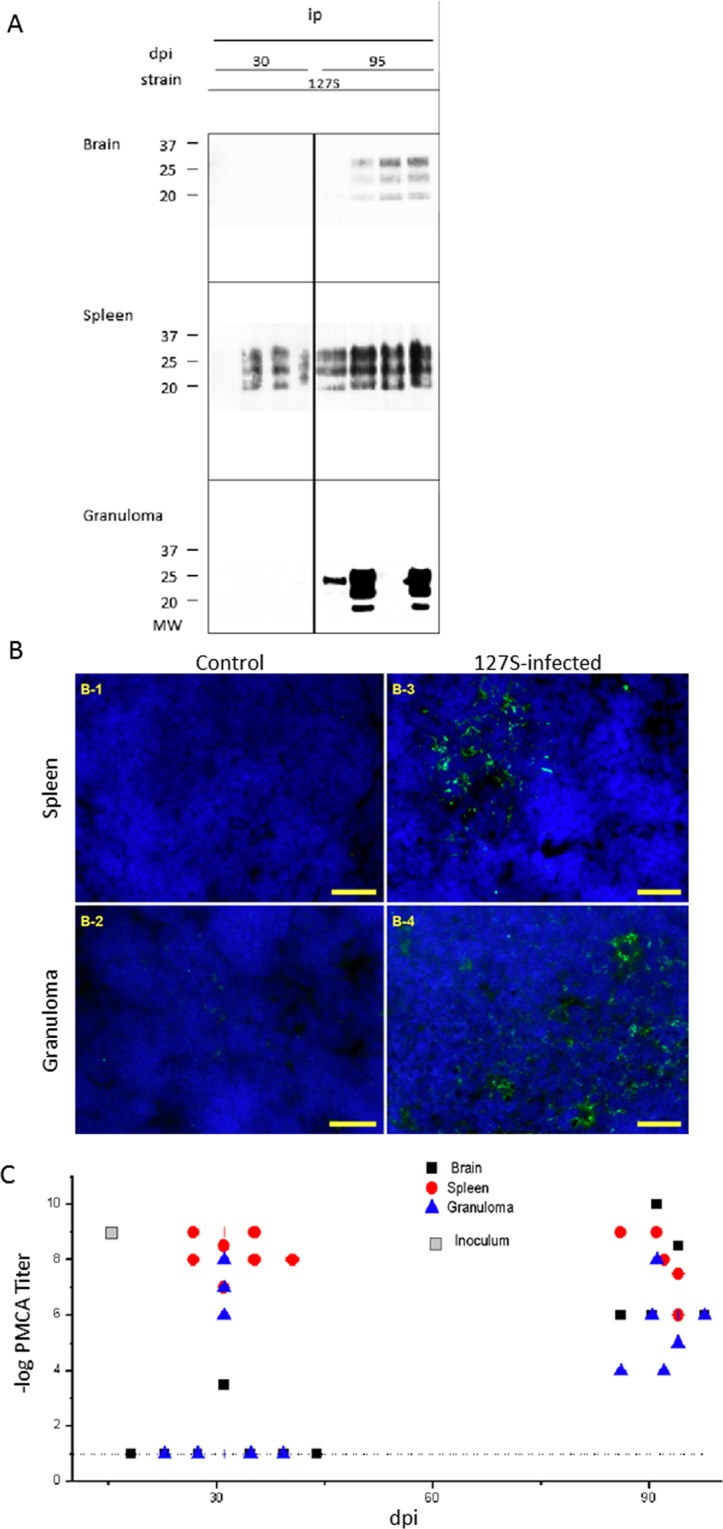
Detection of PrP^Sc^ in the brain, spleen and granuloma of 127S-infected mice. At various times post-infection, groups of animals were sacrificed and brain, spleen and granuloma were removed for tissue processing and analysis. (**A**) proteinase-K digestion followed by western blot analysis using biotinylated Sha31 anti-PrP antibody. Note that the exposure times for brain and spleen WB membranes ranged from 3 to 5 minutes, while the granuloma samples required longer exposure times (over 3 hours). (**B**) Immunofluorescence (IF) detection of PrP^Sc^ in the spleen or granuloma of tg338 mice inoculated with 127S prions. Labeling of PrP^Sc^ in spleens (B-1, B-3) and granulomas lymphofollicular structures (B-2, B-4) from control (B-1, B-2) or infected animals (B-3, B-4). Ten-micrometer-thick slices were cut on a cryostat, fixed in 5% paraformaldehyde, permeabilized in 0.5% X-100 Triton, and treated with 3.5 N guanidinium thiocyanate before the avidin-biotin blocking reaction followed by biotinylated Sha31 incubation and streptavidin-Alexa-488 labeling, slide mounting using fluoromount and acquisition under a mono CCD camera. Images in false colors: blue, DAPI counterstaining; green; biotinylated Sha31, labeling guanidinium-resistant PrP^Sc^. Bars: 20 µm. (**C**) PMCA templating activity of PrP^Sc^ present in individual brains, black ■; spleens, red ●; and granulomas, blue ▲ from 127S-inoculated mice. The log titer (regarded as the last Log_10_-positive dilution) is represented versus time (days post-inoculation). Dotted lines represent the detection threshold. Gray squares represent the inoculum titer. See Supplementary Table 1 for a percentage of positive samples.

As shown in Fig. 3A, 127 S PrP^res^ was detected by western blot analysis in the spleens of all granulomatous tg338 mice at day 30 post-infection (although one of them showed only a barely detectable signal). At this time point, PrP^res^ was detected neither in the brain nor in the granuloma of the infected animals. At day 95, the three tissues contained detectable amounts of PrP^res^. One out of the 4 animals tested showed very weak but positive signals by WB in the brain, and one provided inconsistently positive signals in the granuloma. The PrP^Sc^ was also detected in both spleen and granuloma of the 127 S-infected animals (Fig. 3B).

To improve the detection and titrate the prion-templating activity in the collected tissues, we used the mb-PMCA (miniaturized bead protein misfolding cyclic amplification) method, which is highly efficient for amplifying 127 S prions^25^. Healthy tg338 brain lysates were seeded at serial 10-fold dilutions of the tissue of interest and subjected to one 48-h single round of mb-PMCA. The amplified products were tested for the presence of PrP^res^ by western blotting. The results are summarized in Fig. 3C. For the brain, PMCA-positive reactions were observed in only one brain at day 30. At day 95, all the brains analyzed showed positive reactions. Based on the limiting dilution values, there was an overall increase in the templating activity by 10^3^–10^7^-fold (depending on the brains analyzed) during this period. For the spleen, all the samples analyzed were PMCA-positive and at their maximum seeding activity from day 30 onwards, suggesting a plateau of activity, as previously described^26^. For the granulomas, 3 out of the seven tested samples were PMCA-positive at day 30, with limiting dilution values approaching that found for the spleen, thus suggesting a substantial replicative activity in this tissue. At day 95, all the granulomas showed a positive score with limiting dilution values ranging from 10^−4^ to 10^−8^ (see supplementary table for percentages of PMCA-positive samples).

Collectively, these data indicated that intraperitoneally inoculated 127 S prions could be detected in a proportion of the granulomas, as early as in the spleen. The positive samples exhibited titers comparable to those observed in the spleen. This difference in the number of affected granuloma versus spleen was fully consistent with a previous report conducted in a wild-type mouse model^21^.

### Prion strain-dependent lymphotropism is maintained in the granuloma

**Figure 4:**
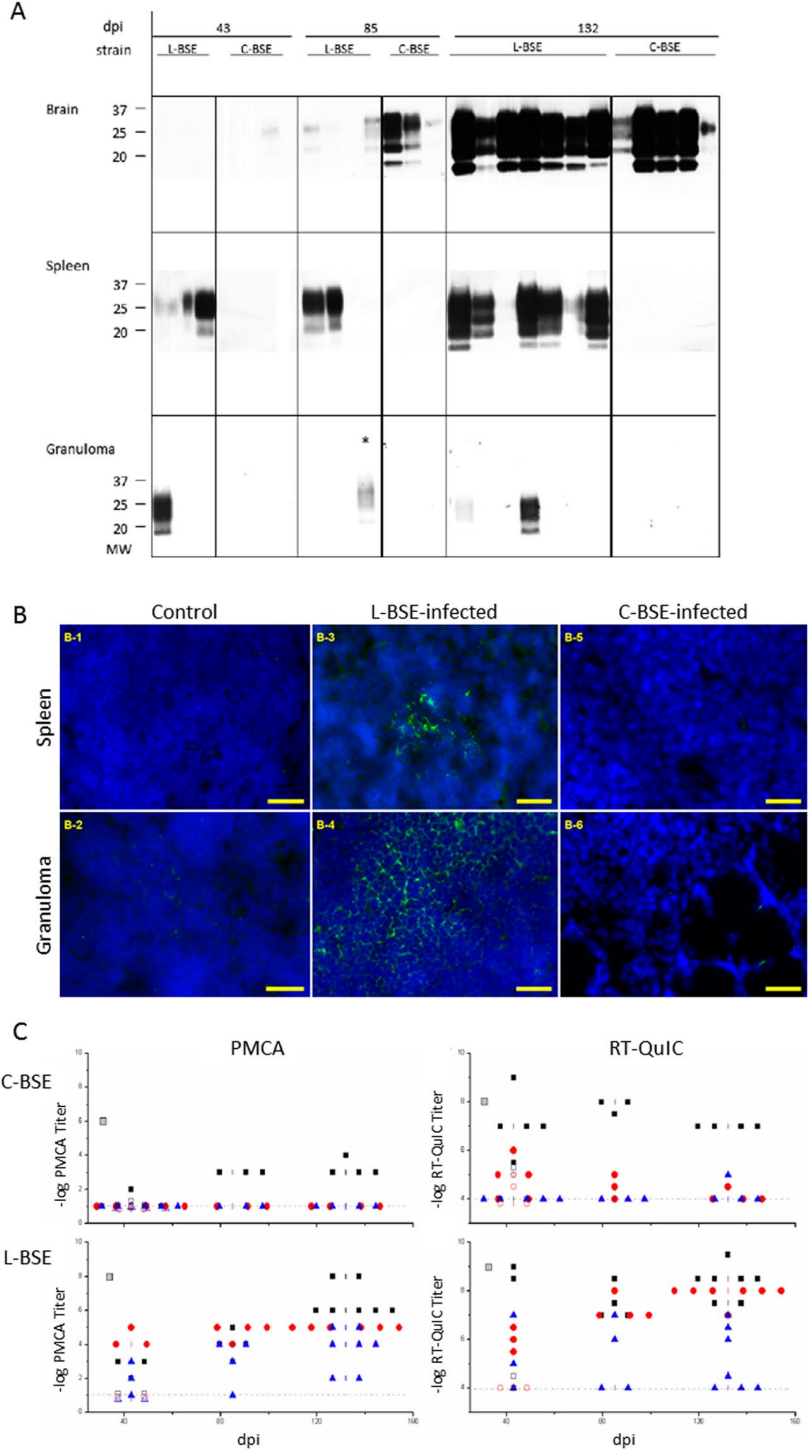
Detection of PrP^Sc^ in the brain, spleen and granuloma of L-BSE or C-BSE-infected mice. At various times post-infection, groups of animals were sacrificed and brain, spleen and granuloma were removed for tissue processing and analysis. (**A**) proteinase-K digestion followed by western blot analysis using biotinylated Sha31 anti-PrP antibody. Note that the exposure times for brain and spleen WB membranes ranged from 3 to 5 minutes, while the granuloma samples required longer exposure times (over 3 hours). (**B**) Immunofluorescence (IF) detection of PrP^Sc^ in the spleen or granuloma of tg338 mice inoculated with L- and C-BSE prions. Labeling of PrP^Sc^ in spleens (B-1, B-3, B-5) and granulomas lymphofollicular structures (B-2, B-4, B-6) from control (B-1, B-2), L-BSE-infected animals (B-3, B-4) or C-BSE-infected animals (B-5, B-6); (**C**) PMCA and RT-QuIC templating activity of PrP^Sc^ present in individual brains, black ■; spleens, red ●; and granulomas, blue ▲, from C-BSE or L-BSE-inoculated mice. Gray squares represent the inoculum titer. Open symbols represent PrP^0/0^ animals. See Supplementary Table 1 for a percentage of positive samples.

We next addressed whether prion strain-specified tropism toward the spleen was also maintained in granulomatous structures by inoculating granulomatous tg338 mice with either C-BSE or L-BSE prions. By western blot analysis (Fig. 4A), L-BSE PrP^res^ was detected in spleens from day 43 onwards, and the levels of accumulation increased until the last stage of disease. As expected, C-BSE PrP^res^ was not detected in the spleens of tg338 mice throughout the time course of disease. A parallel situation was found in the granulomas, which remained constantly PrP^res^-negative in C-BSE-infected animals, regardless of the sampling time point. In L-BSE-inoculated animals, some granulomas were PrP^res^-positive, yet their percentages and levels of PrP^res^ accumulation overall were lower than in the spleen, as observed for the 127 S-infected samples: at day 43 dpi, PrP^res^ was found in the granuloma of one L-BSE-inoculated animal (1/4); one sample was also PrP^res^-positive at 85 dpi (1/4), and only 2/7 showed clear PrP^Sc^ accumulation at 132 dpi.

**Figure 5:**
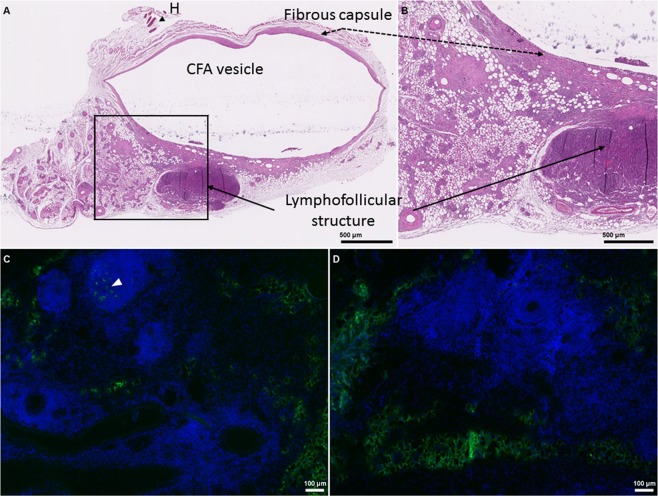
Structural organization of a CFA-induced granuloma and PrP^Sc^ detection in the adjacent lymphofollicular structure. Granulomas were induced in tg338 mice by two successive injections of CFA emulsified in PBS (50% vol/vol) injected subcutaneously into the abdominal or dorsal face of the animals. (**A**), Hematoxylin/eosin staining after formalin fixation and paraffin embedding. Slides were scanned using a Hamamatsu digital NanoZoomer. LFS, adjacent lymphoid structure; H, hair follicles. The marked area delineates the underlying magnified insert (panel B). Panels C and D represent immunofluorescence (IF) labeling of PrP^Sc^ of granuloma sections from 127 S-infected and uninfected control mice, respectively. The white arrowhead highlights the specific labeling of Sha31 within lymphofollicular zones surrounding the granuloma core.

**Figure 6:**
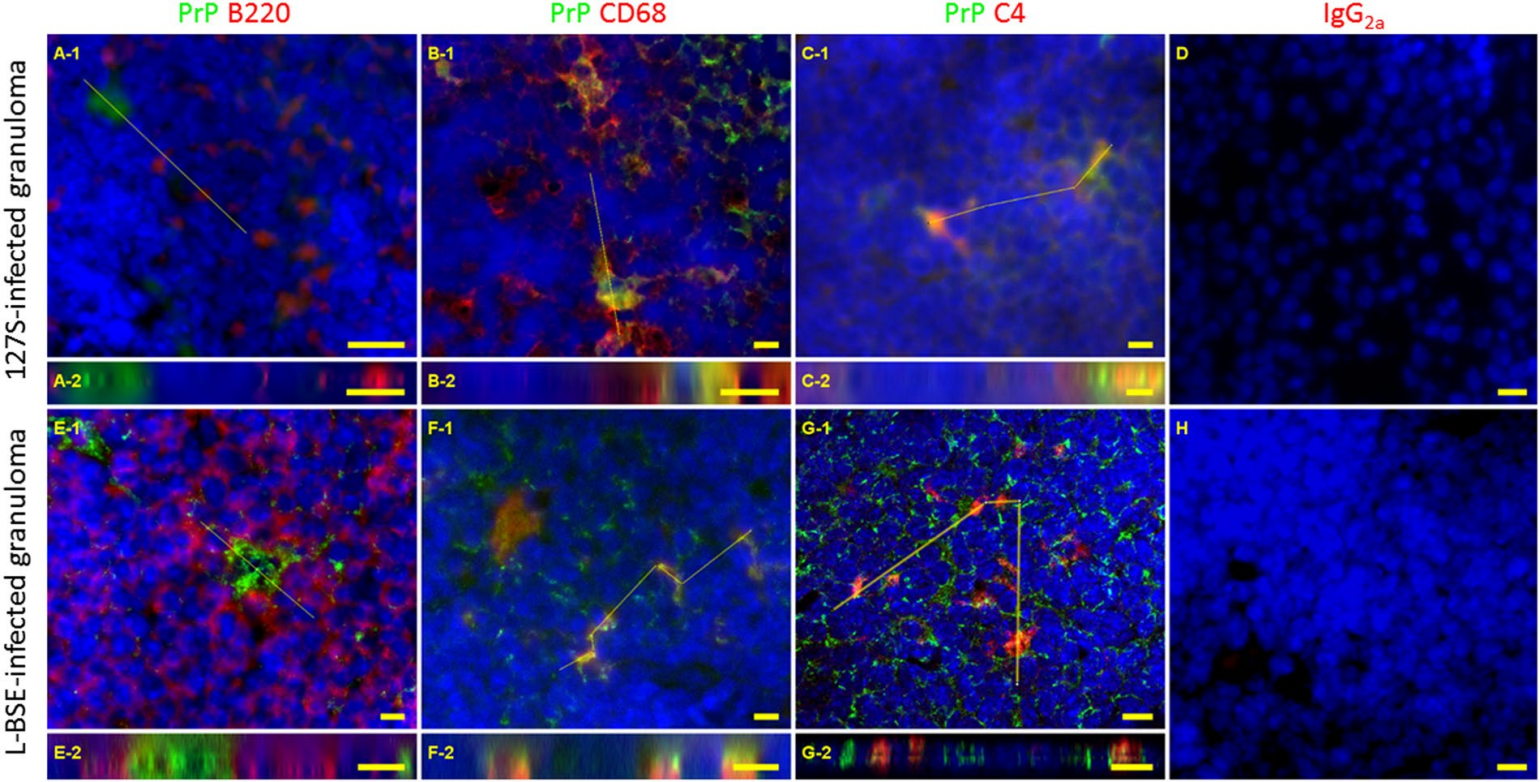
Double immunofluorescent labeling of lymphofollicular structures in granuloma from 127 S- (panels A-D) or L-BSE- (panels E-H) -infected animals. Ten-micrometer-thick slices were cut on a cryostat, fixed in 5% PFA, permeabilized in 0.5% X-100 Triton, and treated with 3.5 M guanidinium thiocyanate, followed by the avidin-biotin blocking reaction. Incubation was performed with biotinylated Sha31 (PrP^Sc^, green) + anti- B220 (panels A and E), anti-CD68 (panels B and F) or anti-C4 (FDC-M2, panels C and G) (red) antibodies, followed by anti-rat-Cy3 + streptavidin-Alexa-488 conjugates, slide mounting using fluoromount and acquisition under a mono CCD camera. Panels D and H correspond to spleen and granuloma sections stained using IgG_2a_ isotype control antibody. Images in false colors: blue, DAPI counterstaining. Bars: 10 µm. Longitudinal panels (A-2 – G-2) represent Z reslicing alongside yellow lines drawn in panels A-1 to G-1, respectively.

These results were confirmed by immunofluorescent detection of PrP^Sc^ on tissue sections. The sections were treated with guanidinium thiocyanate to allow the detection of chaotropic agent-resistant PrP^Sc^ structures^27^. PrP^Sc^ was not detectable in either the spleen or granuloma of control (Fig. 4,B-1,B-2) or C-BSE-inoculated mice (Fig. 4,B-5,B-6, respectively), even at late time points (132 dpi). By contrast, PrP^Sc^ could be detected in both the spleens and granulomas Fig. 4,B-3,B-4, respectively) of L-BSE-inoculated animal, at near disease terminal stage, as observed for the 127 S prions (spleen colonization was effective even at earlier time points (30 and 43 dpi for 127 S and L-BSE strains, respectively, data not shown). Granuloma-positive staining was less frequent but still occurred at these time points (not shown).

The collected tissues were then analyzed by mb-PMCA to further demonstrate the distinct tropism of L-BSE and C-BSE prions for granulomatous structures. Lowly-diluted (10^−1^ dilutions representing the lowest dilution achievable) spleen and granuloma extracts from C-BSE-infected mice did not produce PrP^res^-positive amplicons by PMCA amplification (Fig. 4C). In contrast, PMCA-amplified products seeded with spleen and granuloma extracts from L-BSE-infected mice were mostly PrP^res^-positive from day 43 onwards. However, the efficacy of the mb-PMCA assay to amplify C-BSE vs L-BSE prions appeared discrepant compared to the bioassay. The limiting dilution values achieved with C-BSE brain material were constantly >100-fold lower than with L-BSE brain material, despite similar values by bioassay in indicator tg338 mice (Table 1). Thus, we refined the mb-PMCA analysis with the data obtained using the RT-QuIC (Real Time Quaking-Induced Conversion) assay^28^ to accurately and quantitatively detect the replicative activity of L-BSE and C-BSE prions in central and peripheral tissues. Indeed, using this assay, the limiting dilution values from C-BSE and L-BSE brain materials were much closer throughout the time course of the disease (Fig. 4C). By RT-QuIC, C-BSE granulomas remained negative. A few spleens appeared to be positive above the detection threshold (10^−4^ dilution), in particular at 43 dpi (Fig. 4C). The number of positive samples and the limiting dilution values decreased over time. Such low values were found in PrP knock-out mice inoculated with C-BSE and L-BSE prions (Fig. 4C, open symbols), which suggested that we were detecting the residual fraction of the inoculum that had spilled over into the blood and become trapped in the spleen.

RT-QuIC, like PMCA, confirmed that a proportion of L-BSE granulomas were positive from day 43 onwards (Fig. 4C). The proportion of PMCA (or RT-QuIC)-positive granulomas increased with time, ranging from 66% of positive animals at day 43 to 75% (50% with RT-QuIC) at 85 dpi and 100% (57%) at 132 dpi (see supplementary table for percentages of PMCA- and RT-QuIC-positive samples). The templating activity of the positive granulomas was highly variable, with limiting dilution values ranging from 10^−2^ to 10^−5^. The number of positive granulomas and the templating activity were significantly higher than those found in PrP^0/0^ mice, suggesting the active replication of L-BSE prions in this structure.

Collectively, our results indicated that prion strain-dependent tropism for the lymphoid tissue was preserved in granulomatous structures, as observed in the spleen.

### PrP^Sc^ deposits mainly co-localize with CD68^+^ macrophages and C4^+^ or mfge-8^+^ cells in the granuloma core and the surrounding lymphofollicular structures

While evaluating the structural integrity of the granulomas isolated from the infected mice, we frequently noticed the presence of ovoid adjacent structures filled with small cells and a high nuclear density, observable by hematoxylin/eosin staining of a granuloma sampled from a 127 S-inoculated mouse (see Fig. 5 and Supplementary Fig. 1 for an overview of the main immunofluorescence staining pattern using B, T lymphocytes, macrophages and FDC markers, B220, CD68 and mfge-8 or C2/C4 antigens staining, respectively). Overall, the granulomas lymphofollicular structures were highly reminiscent of the inflammatory tertiary follicles that develop in the surroundings of chronic inflammatory stimuli (see^18^ and^29^ for a review). Interestingly, the strongest specific PrP^Sc^-positive signal was located in these lymphofollicular structures (Fig. 5C, white arrowhead), while no specific staining could be observed in uninfected control animals (Fig. 5D). This particular staining was much more intense than that within the regular granuloma structure, which was particularly difficult to discriminate from the uninfected animals due to the high background levels.

Since the FDC counts in the lymphofollicular structures surrounding the granuloma were not significantly different from those in the spleen (see Supplementary Fig. 2), we next aimed to identify the cell types supporting prion accumulation in these lymphofollicular structures. Spleens were used as controls. Using consecutive slides, we could observe that FDC-M1 antibody also stained cells in close vicinity to the PrP^Sc^-positive cells (data not shown). The antigen recognized by this antibody was seriously damaged by guanidinium treatment, thus precluding the use of FDC-M1 to establish a formal association of PrP^Sc^ and FDCs with a double-staining procedure. Therefore, histological slices from granulomas and spleens collected from mice at various time points post-infection were stained with biotinylated anti-PrP Sha31 and antibodies against the following cellular markers: B cells (B220), macrophages (CD68) and FDCs (C4 complement marker, recognized by FDC-M2 antibody). As anticipated based on a previous report^30^, PrP^Sc^ accumulation in the spleen was mainly located within the germinal center light zone. B220 labeling did not colocalize with the PrP^Sc^ signal in the spleen (not shown), and co-staining was also unnoticeable in the granuloma (Fig. 6, panels A, 127 S infected samples; panels E, L-BSE-infected samples) while C4 as well as CD68 staining were superimposed on the 127 S- (Fig. 6, CD68, panels B; C4, panels C) or L-BSE-PrP^Sc^ signals (Fig. 6, CD68, panels, F; C4, panels G). Z-stacks were generated for the CD68, B220 and C4-labeled slides (Fig. 6, panels A-2, B-2 and C-2) and confirmed that CD68^+^ and C2/C4^+^ cells were close to the 127 S-PrP^Sc^ signals, while B220^+^ cells were clearly not associated with Sha31 PrP^Sc^ staining in the granuloma of 127 S-infected animals. Similarly, multiple labeling studies revealed that L-BSE PrP^Sc^ staining in the lymphofollicular structures surrounding the granuloma was associated with CD68^+^ or C2/C4^+^cells, but not with B220^+^ cells (Fig. 6, panels E-1 to G-2).

**Figure 7:**
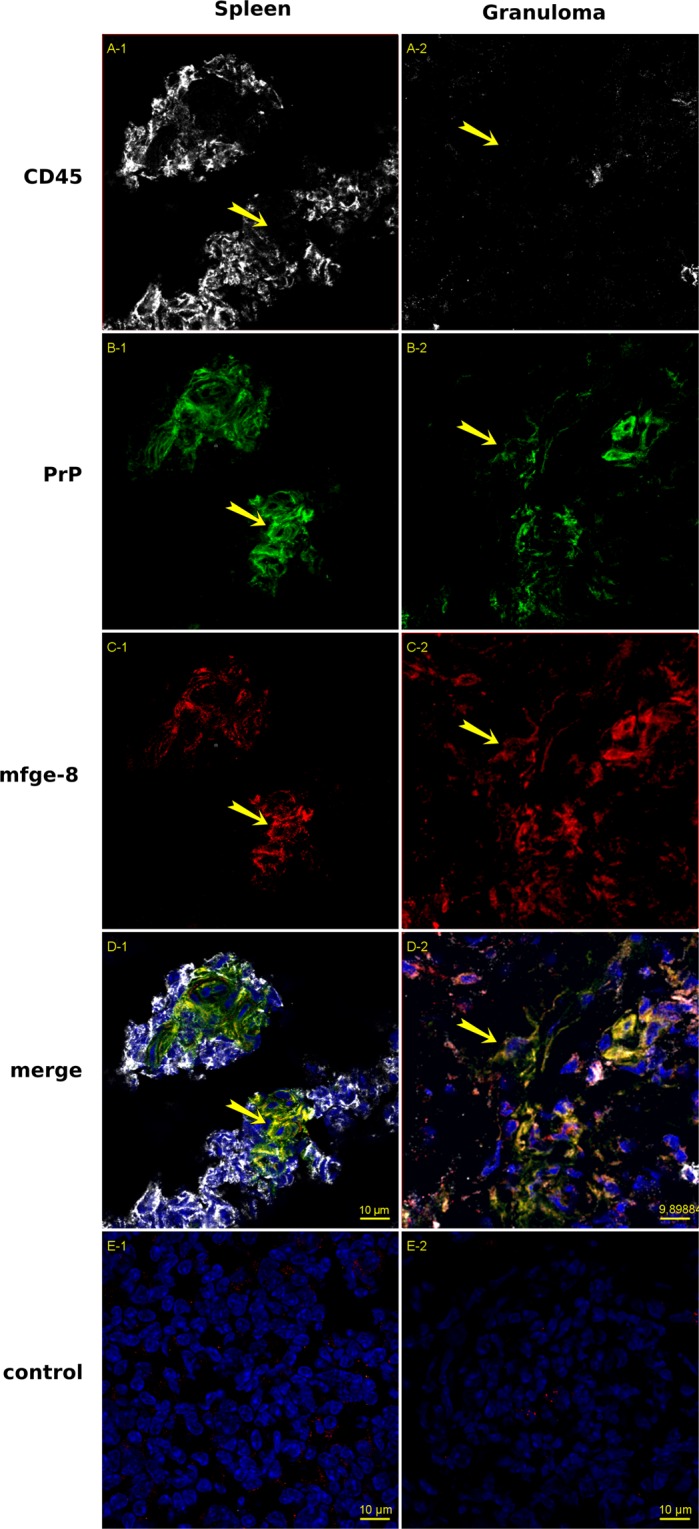
Triple immunofluorescence labeling of Spleen (panels 1) and Granuloma sections (panels 2) from 127 S-infected animals. Nine-micrometer-thick slices were cut on a cryostat, fixed in 5% PFA, permeabilized in 0.5% X-100 Triton, blocked with 5% Bovine serum albumin and 2% rat and mouse sera, followed by the avidin-biotin blocking reaction. Incubation was performed with antibodies against CD45 (rabbit anti CD45 serum, gray, panels A) PrP^C^ (biotinylated Sha31, green, panels B), and mfge-8 (FDC-M1 rat monoclonal antibody, red, panels C). Incubation was followed with, anti-rat-Cy3 + streptavidin-Alexa-488 + anti-rabbit-Alexa 645 labeling, slide mounting using fluoromount and acquisition under a mono CCD camera. Panels D1 and D2 show the merged image built with the 4 channels (images in false colors: blue, DAPI counterstaining). Panels E1 and E2 correspond to spleen and granuloma sections stained using IgG_2c_ isotype control and secondary antibodies as staining control. Bars: 10 µm. Yellow arrows show PrP^+^/mfge-8^+^ cells that do not stain positive for CD45.

Considering that the conditions were not optimal for the rigorous characterization of supposed FDCs in the granulomas (guanidinium treatment, non-specific FDC-M2 antibody, etc), we needed to get further convinced that the PrP^+^/mfge-8^+^- or PrP^+^/C2/C4^+^ cells were indeed follicular dendritic cells (ie differentiated stromal cells), not just macrophages (ie lymphoid cells) that could have loaded prion particles and mfge-8 antigens as well. For that purpose, a general lymphoid marker should discriminate between stromal and lymphoid cells. Therefore, spleen and granuloma sections sampled from a 127 S-infected animal were triple-labeled with antibodies recognizing CD45, mfge-8 and PrP. Preservation of mfge-8 antigen precluded the use of guanidinium isothiocyanate, so PrP labeling mainly identified the cellular antigen of the prion protein, not the misfolded conformer. The resulting tri-labeled slides were observed under a confocal inverted microscope (Fig. 7). As expected from the literature and our double-label study, we were able to detect PrP^C^ positive cells decorated with mfge-8 in the germinal centers of the spleen. These cells did not stain for CD45, as for true conventional splenic FDCs. When granuloma sections were studied, some cells positive for PrP and mfge-8 were also stained by CD45, establishing that they belonged to the lymphoid lineage. But we also could detect PrP^C+^/mfge-8^+^ cells that did not stain positive for CD45 (Fig. 7 panel 2). This finding strengthened the odds that there were indeed true FDCs in the granulomatous environment. Both types of cells could be observed in the same microscope field (see Supplementary Fig. 3A). Spatial fluorophore distribution within the tissue was further investigated (see Supplementary Fig. 3B). These observations confirmed that both PrP and mfge-8 labeling could be observed in the absence of neighboring CD45 (see individual and merged channels in yellow-boxed of Supplementary Fig. 3b showing slicing along the yellow line in both spleen and granuloma). Surprisingly, triple –labeled cells could hardly be detected in the spleen of the infected mice: at least, the mfge-8^+^/CD45^+^ cells did not stain for PrP.

In conclusion, both the spleen and granuloma contained macrophages and FDCs, with which misfolded PrP could be associated when mice were inoculated with lymphoid tissue tropic prions, either 127 S scrapie or L-BSE adapted to ovinized mice. Furthermore, we observed that the cellular organization surrounding the granuloma was reminiscent of tertiary lymphoid organs. These structures displayed higher PrP^Sc^ signals than the granuloma core.

## Discussion

Here we provide evidence that the strain-dependent prion LTT is mostly preserved between the spleen and inflammatory, granulomatous structures from infected mice. Both LTT prions studied herein replicated in the spleen and in the granuloma of the infected animals, while the non-LTT prion did not. We further showed that prion accumulation in the granuloma might preferentially proceed through adjacent lymphofollicular structures, suggesting that the lymph node environment is much more adapted to the prion accumulation/replication than the regular inflammatory granuloma structure.

Certain prions can actively replicate in the lymphoid tissue of infected animals. Although this step is not mandatory, it allows their efficient routing to the CNS after extraneural infection^31,32^. By contrast, other prions are not detected within the lymphoreticular system, although we can reasonably suspect that they nevertheless circulate at very low levels through these structures. The determinants of prion tropism toward specific brain regions and lymphoid tissue are mostly unknown but are strain-specific. The adaptation of atypical L-BSE prions to ovine PrP tg338 mice led to the individualization of a prion closely resembling classical C-BSE prions in terms of incubation time, PrP^res^ electrophoretic profile and resistance to proteolytic digestion, neuroanatomical deposition of PrP^res^ and vacuolation, and infectious titer (^24^ and unpublished). Despite these phenotypic similarities, the two agents showed marked dissimilar tropisms for the lymphoid tissue, with the C-BSE agent losing its ability to propagate efficiently in the spleen upon iterative passage. Beyond suggesting that minimal conformational differences between PrP^Sc^ conformers (which were not possible to observe by any available conventional strain typing method, such as guanidinium denaturation or proteolytic digestion^33^) could result in strikingly different lymphotropism, these two agents represented an ideal experimental paradigm to further study host requirements for lymphoid replication.

Within lymphoid tissues, FDCs are believed to be the main site of prion replication. When a former publication found that prions could accumulate in granuloma supposedly devoid of FDCs^21^, the question arose concerning whether those structures would share the same kind of prion strain-dependent selectiveness with the spleen despite an apparently distinct prion-permissive cell type. We induced subcutaneous granulomas in tg338 mice and challenged them with prion strains differing in their lymphoid tropism. The time course of spleen and granuloma contamination was superimposable for the LTT 127 S scrapie prion and the other LTT agent L-BSE. However, both strains replicated at lower and more variable levels in the granuloma than in the spleen. In marked contrast, the non LTT C-BSE prion was not detected in any of these structures, even after *in vitro* prion amplification by mb-PMCA^25^. Using RT-QuIC as another cell-free assay with superior sensitivity for C-BSE prions, a few granulomas and spleens provided positive scores, yet the proportion and limiting values were consistent with the inoculum remanence, as evidenced in PrP^0/0^ mice^28^. There was therefore a 3-4 orders of magnitude difference in splenic or granuloma prion titers (obtained using either PMCA or RT-QuIC) between L- and C-BSE despite a similar titer in the brain. Thus, the L-BSE that was adapted to ovinized mice was a true LTT prion, while the classical C-BSE was not.

Former studies have established that FDCs are the first extraneural cell type that accumulate prion and are a necessary cell type for efficient prion infection^11–15,34^. Granulomas have been described as inflammatory structures capable of prion accumulation that are devoid of FDCs. However, Maestrale and colleagues showed that PrP^Sc^ deposition in inflammatory structures of sheep naturally infected with scrapie required a lympho-follicular environment and probably FDC-like cells^17^. Accordingly, naturally developed granulomatous lesions were not fully suitable environments for the detection of prions. qRT-PCR experiments supported the view that the local cellular granulomatous environment did not provide the appropriate cytokines (lymphotoxins-α, -β, TNF, CXCL13, CCL19, CCL21, CxCR4 and CxCR5) required for lymph node development and maintenance of FDCs.

Macrophages have been described to play a particularly important role in the spleen of infected animals, where they can capture and sequester a significant amount of PrP^Sc^^35^. Here, CD68^+^ cells were found in the general core of the granuloma, and these cells were also positive for PrP^Sc^. This observation indicated that the main granuloma PrP^Sc^ positive signal could be due to macrophage scavenging activity. Both LTT prions used in this study (127 S and L-BSE) could be detected in the CD68 ^+^ macrophages of the granuloma core, but that was not the case with C-BSE prion. This observation suggested that the scavenging activity of the macrophage is somehow associated to the local production or accumulation of PrP^Sc^ in the granuloma.

Surprisingly, our data established that lympho-follicular structures could develop adjacent to the canonical granulomatous structure that we induced in mice following Freund’s adjuvant injection. These structures contained small cells with a highly reduced cytoplasmic compartment. In addition, immunofluorescence labeling identified B220^+^, CD68^+^ and mfge-8^+^ and C2/C4^+^ cells. We were also able to observe PrP^Sc^ deposition, which could additionally co-localize with CD68^+^ cells, but not with B220 antigen. Though not completely specific for FDCs, mfge-8 and C2/C4 antigens are good markers and commonly used for the identification of these stromal cells in the lympho-follicular environment^30,36^. Although a combination of these markers with several other markers of stromal origin (CD54, CD106, podoplanin, clusterin and PrP^C^^37^ and see^38^ for a review), they are the most specific markers for the identification of FDCs in the context of IF or IHC staining, especially if they are combined with the use of CD45 as an exclusion marker for the Stromal/Lymphoid cells discrimination. A definitive element in favor of the presence of true FDCs would however require the experimental presentation of immune complexes following peripheral or iv administration of fluorescent antigens^39^.

Granulomas induced with complete Freund’s adjuvant seemed to induce the ectopic development of nearby lymphofollicular structures. However, these structures were not observed in every microscopic section studied, which might be. This is to be related to the fact that PK-resistant PrP^Sc^ signals and prion seeding activity were absent in a number of these structures throughout the disease pathogenesis of 127 S or L-BSE prions. These negative granulomas could correspond to dissected areas that were sampled without the surrounding lympho-follicular structures. Unfortunately, we could not formally link the prion-positive status of the granuloma with these ectopic structures, since each sample could not be used for both immunohistochemistry and western blot/cell-free assays. In addition, IF prion detection in the case of L-BSE was difficult to confirm in the granuloma core. Therefore, it would be interesting to focus on the distinct cellular organization of the granuloma structure in relation to the prion strains: infections with RML and 127 S scrapie strains (respectively used in^21^ and the present study) resulted in massive granuloma staining, while L-BSE prion detection was apparently more restricted to lymphoid-like structures. Consequently, lymphocompetence should be regarded as a continuous criterion, particularly in an inflammatory context. This phenomenon is of public health importance when considering vCJD as a lymphocompetent prion that can also replicate in various organs and benefit from favorable conditions in the context of concurrent chronic inflammatory diseases^40^.

## Materials and Methods

### Ethics statement

All animal experiments were performed in strict accordance with EU directive 2010/2063 and were approved by the local ethics committees of the authors’ institutions (Comité d'éthique appliqué à l’expérimentation animale, Comethea, permit number 12/034 and 12/127).

### Transgenic mouse lines and prion strains

The ovine PrP tg338 mouse line (Val_136_Arg_154_Gln_171_ allele) is homozygous and overexpresses approximately eight-fold the heterologous PrP^C^ on a mouse PrP null background. PrP^0/0^ mice were kindly provided by C. Weissmann (Zürich I line^41^). Mice were maintained under specific pathogen-free (SPF) conditions until the infection or inoculation procedures. Then, the mice were housed in a biosafety level 3 laboratory.

The 127 S scrapie prion strain was obtained through serial transmission and subsequent biological cloning by limiting dilutions of the PG127 field scrapie isolate from Tg338 mice^23^. The 127 S infectious titer was 10^9.2^ LD_50_/g of tg338 brain^42^. Pools of 127 S sick tg338 mouse brains were prepared as 20% (wt/vol) homogenates in 5% glucose using a tissue homogenizer (Precellys 24 Ribolyzer, Ozyme, Bertin technologies, France).

L-BSE and C-BSE prions were obtained through serial transmission (5 passages) of cattle classical and atypical L-type BSE in tg338 mice (see Table 1).

### Induction of granulomatous structures in tg338 mice and prion infection

Tg338 mice were inoculated subcutaneously with 100 µL of 50% Complete Freund’s adjuvant CFA according to a previously described protocol^21^. The injection was performed either on the abdominal or on the dorsal side of mice and was repeated twice in 21 days between each injection. Visible swelling was observed or detected by eye or by local palpation of the injection site.

The granulomatous tg338 mice were inoculated intraperitoneally with 100 µL of a 2% (w/v) 127 S brain homogenate or intracerebrally with 20 µL of a 10% (w/v) C-BSE or L-BSE brain homogenate. PrP^0/0^ mice were inoculated with the same dose. Uninfected control mice were intracerebrally inoculated with the same dose of uninfected brain homogenate. Brains, spleens and granulomas were harvested at selected time-points post-infection, at 30 and 95 dpi for 127 S and at 43, 85 and 132 dpi for L-BSE and C-BSE prions, for which the incubation times were longer than for the former scrapie strain. For PrP^res^ detection by immunoblot, PMCA amplification and RT-QuIC analysis, the collected tissues were homogenized with a tissue homogenizer (Precellys 24 Ribolyzer, Ozyme, Bertin technologies, France) and adjusted to 20% (w/v) in 5% glucose (alternatively, 10% homogenized suspensions were realized for spleen samples or when the granulomas were too small). The samples were stored at −80 °C. For immunofluorescence studies, spleens and granulomas were immediately embedded in O.C.T medium (Fontenay-sous-bois- France) and snap frozen on dry ice before further storage at −80 °C. For histological studies, spleen, granuloma and brain samples were fixed in 4% formalin solution before embedding in paraffin.

### PK treatments for PrP^res^ detection

Brain, spleen and granuloma homogenized samples were treated with PK (final concentration of 115 µG/mL) for 90 min at 37 °C. Loading Buffer (2 × : 50 mM Tris, pH 6.8, 2% SDS, 0.01 bromophenol blue, 2 mM dithiothreitol 50% glycerol) was added, and the samples were denatured at 100 °C for 5 minutes.

### Miniaturized bead-Protein Misfolding Cyclic Amplification (mb-PMCA)

The mb-PMCA procedure has been described in a previous work^25^. In brief, mouse brain lysate from uninfected tg338 mice (10% brain lysate in cold PMCA buffer: 50 mM Tris-HCl pH 7.4, 5 mM EDTA, 300 mM NaCl, 1% Triton-X-100) were used as substrate for 127 S scrapie, L-BSE and C-BSE prion strains. PMCA was performed in a final volume of 36 µL of lysate per well in 96-well PCR microplates (Axygen, Union City, CA, USA). Each well was first filled with one 2.381-mm-diameter Teflon bead (Marteau et Lemarié, Pantin, France). A 4-μL aliquot was serially diluted in the 36 µL-containing wells. The microplate was sealed and placed on a plexiglass rack designated for the cup horn of the Q700 sonicator (Misonix, Farmingdale USA) and subjected to 96 cycles of 30 sec of sonication at 220–240 W power followed by 29 min and 30 s of incubation at 37 °C. After PMCA, the microplates were removed, and 10-µL aliquots of each sample were PK-digested as previously described^25^. The samples were then stored at −20 °C and subjected to dot blot and western blot analyses.

### RT-QuIC

RT-QuIC amplifications were performed as previously described^28,43^. Briefly, the inocula were serially diluted in phosphate-buffered saline (PBS), pH 7.4 containing 0.01% SDS and 1/100 N2 supplement (Thermo Fisher cat# 17502048). Then, 2.04 µL of each dilution was loaded in triplicate in individual wells of a black 96-well optical bottom plate containing 100 µL of PBS mix (10 µM thioflavin T, 1 mM EDTA and 100 µG/mL of recombinant ovine PrP, V136-R154-Q171 allele)^44^. The plate was sealed using Nunc Amplification Tape (Nalgene Nunc International Cat# 232702), placed in a Xenius XM spectrofluorometer (Safas, Monaco) and incubated for 48–60 h at 47 °C. One minute of orbital shaking (600 rpm) was applied every minute, and the fluorescence was recorded every 30 minutes. Data were treated using the 2^nd^ derivate for detection of the inflection timepoint. The sample titer was estimated as the last dilution before the inflection timepoint get across the background level (determined as the inflection timepoint calculated based on the unseeded control).

### SDS-PAGE and western blot analysis

PMCA samples were run on Criterion XT 12% Bis-Tris precast gels (Bio-Rad, Hercules, CA, USA), electrotransferred onto nitrocellulose membranes (Bio-Rad) and probed with biotinylated Sha31 anti-PrP monoclonal antibody^45^. Streptavidin-peroxidase incubation and ECL detection were achieved as described for the dot blot assay. When necessary, the PrP^res^ content of PMCA samples was determined with ImageLab software after acquisition of the signals with a Chemidoc digital imager (Bio-Rad, USA).

### Histochemistry and immunofluorescence

For histochemistry, brain spleen and granuloma samples of C-BSE and L-BSE-infected mice and uninfected control mice with granulomas were fixed with freshly prepared 4% formalin before being embedded in molten paraffin and stored at room temperature until ready for sectioning. Using a microtome, embedded spleen and granuloma tissues were cut into 5-µm-thick sections and mounted on Superfrost Plus slides. The sections were then stained with hematoxylin/eosin.

For immunofluorescence, frozen spleens and granulomas were cut on a cryostat machine (Thermo Fisher scientific). The 7–10-µm-thick sections were then adhered on Superfrost glass slides (Thermo Fisher scientific) and dried under air before storage at −80 °C for subsequent treatments. The tissue sections were fixed with 5% paraformaldehyde (PFA) for 45 minutes and then rinsed with 0.01 M phosphate-buffered saline (PBS), saturated and permeabilized with 5% bovine serum albumin (BSA) and 0.5% Triton-X100 for approximately 1 hour, followed by a 10-minute incubation with each component of the Avidin-Biotin blocking kit (Zymed, Invitrogen) according to the manufacturer’s instructions. The sections were denatured with 3–3.5 M guanidinium thiocyanate and sufficiently washed with 1X PBS followed by double staining by incubation with two primary antibodies: 1/2000 dilution of biotinylated Sha31 mouse monoclonal anti-PrP (Sha31b) and one of the various cellular antigens (rat monoclonal antibodies): FDC-M1 (recognizing mfge-8 antigen, BD Bioscience-Pharmingen, 1/30) and FDC-M2 (recognizing C2/C4 antigen, AMS biotechnology, UK, 1/100) for follicular dendritic Cells, B220 (Invitrogen, 1/200) for B cells, CD3 (AbD Serotec, 1/200) for T cells, and CD68 (Hycult, 1/500) for macrophages, for 1 h at room temperature under saturated humidity. The sections were then washed and incubated with the secondary conjugates streptavidin-Alexa-633 or Alexa-488 (Invitrogen, 1/500) and CY3-donkey anti-rat (Jackson Immunoresearch, 1/500) for 1 h. After 3 washes, the nuclei were stained with DAPI (Sigma, 1/5000). The slides were covered with Fluoromount medium (Interchim, Montluçon, France) and glass lamella before sealing (twice) with nail polish. Subsequently, the slides were decontaminated with 1 N NaOH or bleach water at 6° for 15 min before being evaluated under an inverted epifluorescence microscope (Zeiss Axio Observer Z1) coupled to a mono CCD Pvcam HQ2 camera.

Confocal microscopy was performed with a Zeiss LSM 700 microscope (Confocal facilities, MIMA2 Platform, INRA Jouy-en-Josas, France, https://www6.jouy.inra.fr/mima2) equipped with CLSM 700 confocal laser scanning software, using a Zeiss confocal microscope (images in false colors: blue, DAPI counterstaining) and a Plan Neofluar x40 (NA 1.3(fig A) or a Plan- Apochromat (NA 1.4) × 63 oil-immersion objectives and the 488-, 546- or 645-nm excitation wavelengths of the laser. All images were analyzed using ImageJ 1.51j8 software (Schneider *et al*., 2012) (http://rsb.info.nih.gov/ij/).

## Supplementary information

Supplementary Information
