# Supplementary material for: Prion strain-dependent tropism is maintained between spleen and granuloma and relies on lymphofollicular structures
Source: Sci Rep. 2019 Oct 10;9:14656. doi: 10.1038/s41598-019-51084-1 (PMC6787085; doi:10.1038/s41598-019-51084-1)

# Prion strain-dependent tropism is maintained between spleen and granuloma and relies on lymphofollicular structures

Iman Al-Dybiat^1^, Mohammed Moudjou^1^, Davy Martin^1^, Fabienne Reine^1^, Laetitia Herzog^1^, Sandrine Truchet^1^, Patricia Berthon^2^, Hubert Laude^1^, Human Rezaei^1^, Olivier Andréoletti^3^, Vincent Béringue^1*^, Pierre Sibille^1*^

^1^VIM, INRA, Université Paris-Saclay, 78350, Jouy-en-Josas, France,

^2^UMR Infectiologie et Santé Publique, 37380 Nouzilly, France

^3^UMR INRA ENVT 1225, Interactions Hôtes Agents Pathogènes, Ecole Nationale Vétérinaire de Toulouse, 23 Chemin des Capelles 31076 Toulouse, France

* Corresponding authors; [pierre.sibille@inra.fr](mailto:pierre.sibille@inra.fr); [vincent.beringue@inra.fr](mailto:vincent.beringue@inra.fr)

## Supplementary Information


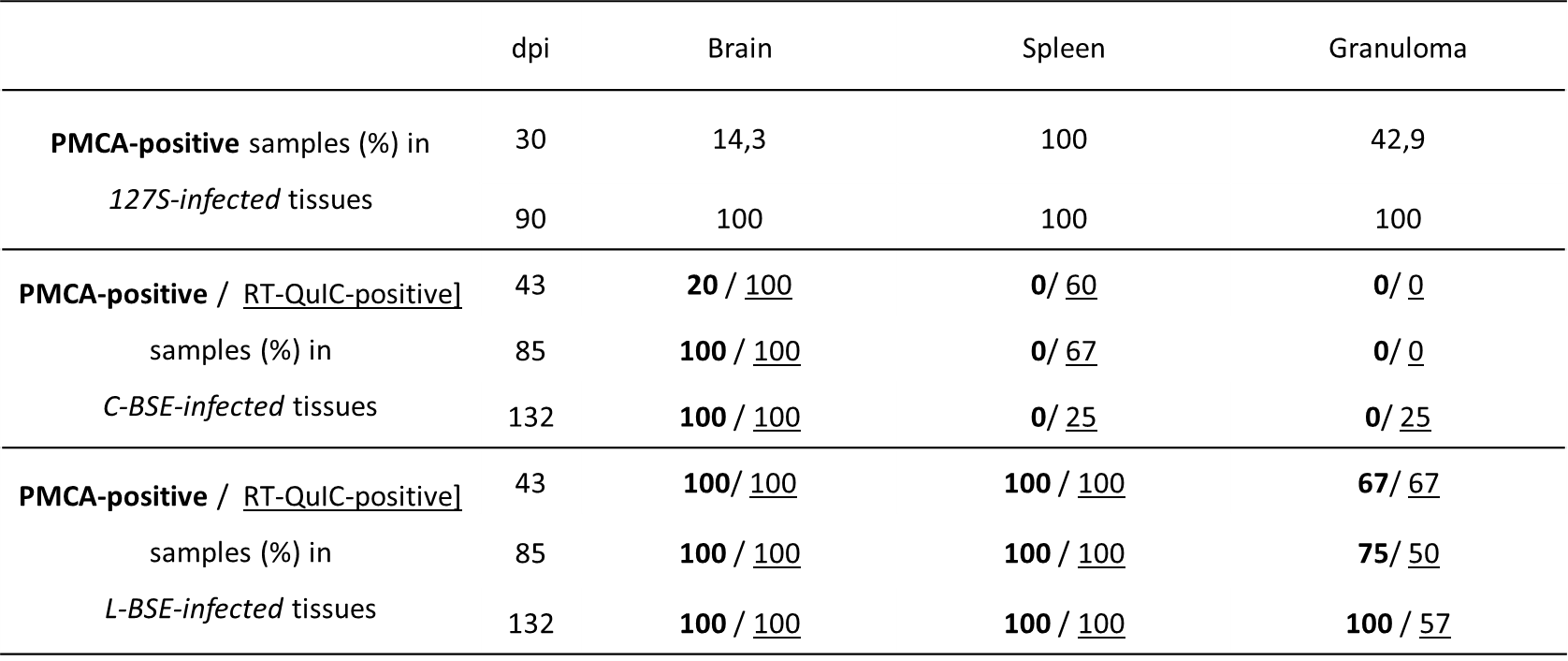
Supplementary Table

Percentages of positive Brain / Spleen / Granuloma samples from mice infected with 127 S , C- or L-BSE prions, sacrificed at the mentioned dpi, and tested using either **PMCA (bolded values)** or RT-QuIC (underlined values). The data (see figures 3-C and 4-C) were considered positive when –log titers were above 1 (PMCA) or 4 (RT-QuIC).

Supplementary Figure 1. Hemalun and eosin staining of regular granuloma and lymphofollicular structures. Typical immunofluorescence staining of usual immune cells markers.

Cryo sections (X5 magnification) representative of regular (upper panel) and lymphofollicular (lower panel) structures were additionally stained using usual cell surface markers (B220, CD90, CD68, vimentin, Ly6G/6C), in addition to more FDC-specific antibodies (FDC-M1 and FDC-M2). Upper panel gathers typical immunofluorescent stainings of the regular granuloma structures. Lower panel represents typical immunofluorescent acquisitions from sections containing lymphofollicular structures.

Supplementary Figure 2 : Median cell density (expressed as cells.mm^-^²) for A/ FDC-M1^+^ and B/ FDCM2^+^ cells in spleen and lymphofollicular structures adjacent to granulomas.

Cell counts were performed on X40 frames (0.0352mm² area) that have been single stained according to the method described in the technical section (without guanidinium treatment). The number of analyzed frames ranged from 8 to 25 per condition. Mann-Whitney non parametrical statistics were performed.

Supplementary Figure 3. Spatial distribution of immunofluorescence in Spleen and Granuloma sections from 127 S-infected animals after anti-CD45, -PrP and -mfge-8 antibody staining.

Nine-micrometer-thick slices were cut on a cryostat, fixed in 5% PFA, permeabilized in 0.5% X-100 Triton, blocked with 5% Bovine serum albumin and 2% rat and mouse sera, followed by the avidin-biotin blocking reaction. Incubation was performed with antibodies against CD45 (rabbit anti CD45 serum, gray) PrP^C^ (biotinylated Sha31, green), and mfge-8 (FDC-M1 rat monoclonal antibody, red), followed with anti-rat-Cy3 + streptavidin-Alexa-488 + anti-rabbit-Alexa 645 labeling, slide mounting using fluoromount and acquisition under a mono CCD camera. Z-stacks (14-18 slices) were acquired for each fluorescence channel using a Zeiss LSM 700 confocal microscope (images in false colors: blue, DAPI counterstaining) and a Plan Neofluar x40 (NA .objective (fig A) or a Plan- Apochromat (NA 1.4) ×63 oil-immersion objectives, fig. B). Reslicing was performed using ImageJ software along the white and yellow lines shown in the 2D upper panels. Reconstruction of slice is presented in the 4 underneath panels (yellow-boxed for the yellow line, white-boxed for the white line): merged image, followed by CD45, PrP and mfge-8 antigen labeling, respectively.


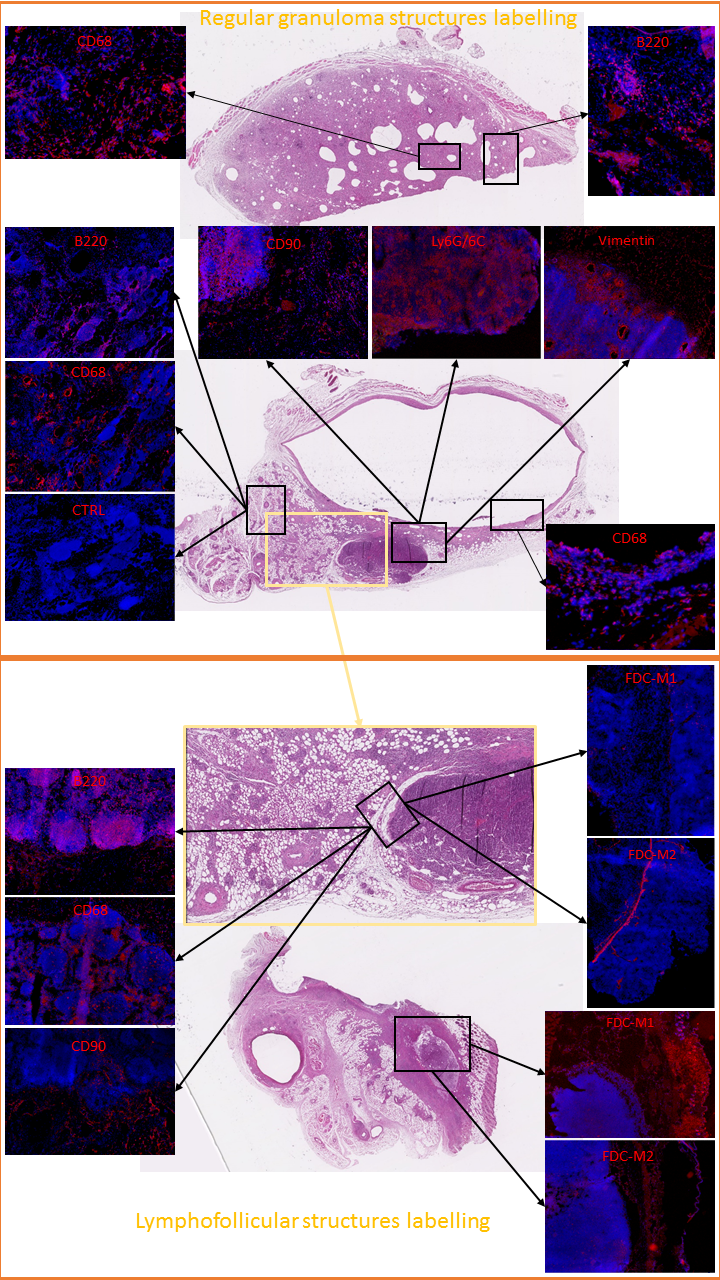



A

A





B


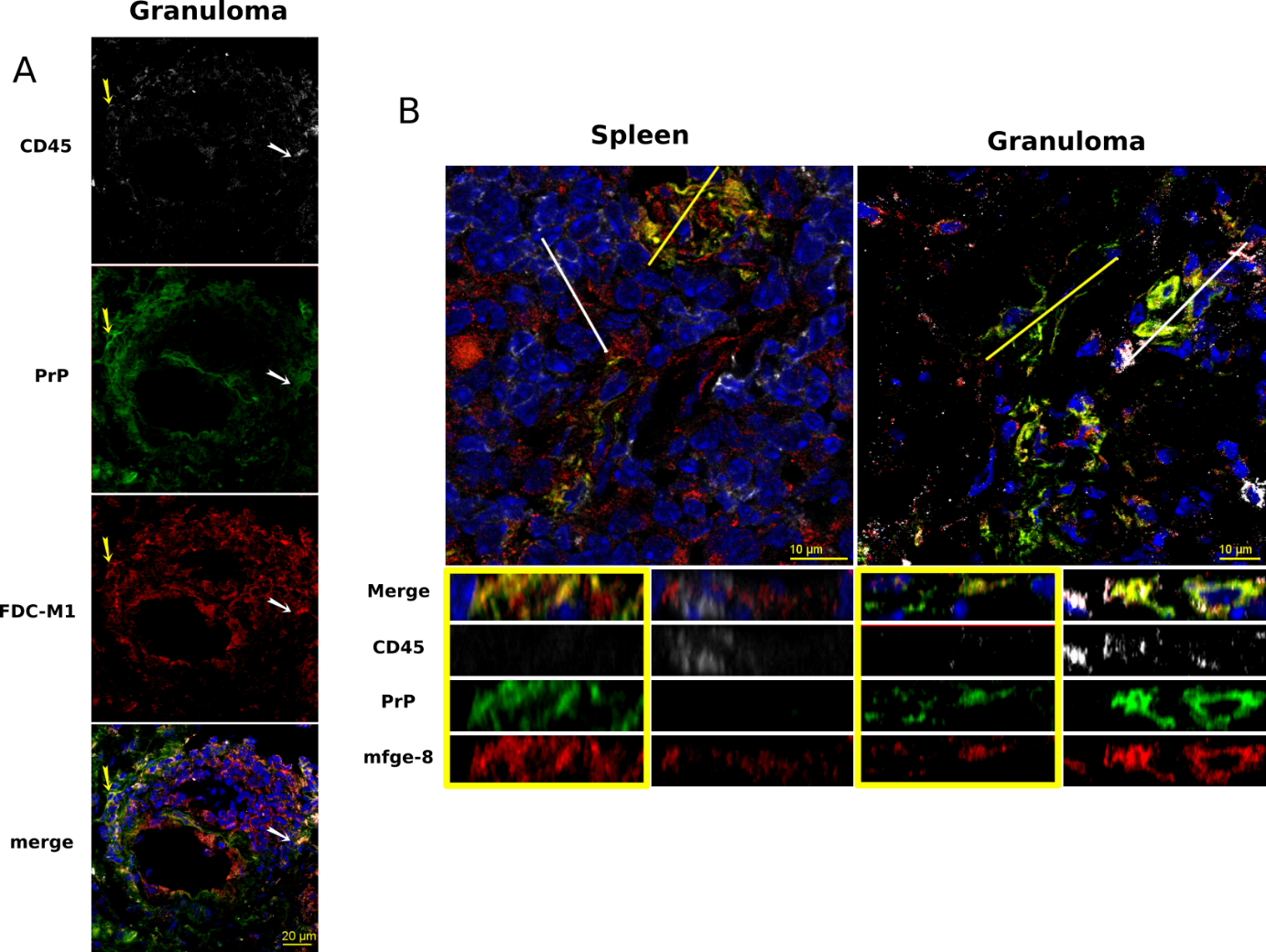

Supplement: Supplementary file 1 — Supplementary Information [file 41598_2019_51084_MOESM1_ESM.docx]
